# Tissue factor in tumor microenvironment: a systematic review

**DOI:** 10.1186/s13045-014-0054-8

**Published:** 2014-08-01

**Authors:** Xiao Han, Bo Guo, Yongsheng Li, Bo Zhu

**Affiliations:** 1 Institute of Cancer, Xinqiao Hospital, Third Military Medical University, Chongqing 400037, PR China; 2 Harvard Institutes of Medicine, Department of Anesthesiology, Center for Experimental Therapeutics and Reperfusion Injury, Perioperative and Pain Medicine, Brigham and Women’s Hospital and Harvard Medical School, Boston 02115, MA, USA; 3 Biomedical Analysis Center, Third Military Medical University, Chongqing 400038, China

**Keywords:** Tissue factor, Tumor microenvironment, Venous thromboembolism, Microparticles, Coagulation

## Abstract

The aberrant hemostasis is a common manifestation of cancer, and venous thromboembolism (VTE) is the second leading cause of cancer patients’ mortality. Tissue factor (TF), comprising of a 47-kDa transmembrane protein that presents in subendothelial tissues and leukocytes and a soluble isoform, have distinct roles in the initiation of extrinsic coagulation cascade and thrombosis. Laboratory and clinical evidence showed the deviant expression of TF in several cancer systems and its tumor-promoting effects. TF contributes to myeloid cell recruitment in tumor stroma, thereby remodeling of tumor microenvironment. Additionally, the number of TF-positive-microparticles (TF^+^MP) from tumor origins correlates with the VTE rates in cancer patients. In this review, we summarize our current understanding of the TF regulation and roles in tumor progression and clinical complications.

## Introduction

Tissue factor (TF), which consist of a 47-KDa-glycoprotein consisting of 263 amino acids (aa) (also named full-length TF (flTF) factor III, thromboplastin, or CD142) and an alternatively splice isoform, are encoded by *F3* gene. The *F3* gene locates on chromosome 1p22-p21 and contains 6 exons that produce a precursor protein with 294 amino acids. After posttranscriptional modification, the functional structure of precursor turns out to be a sausage shape membrane protein consisting of an extracellular domain (219 aa), a transmenbrane residue (23 aa) and a cytoplasmic part (21 aa) [1]. flTF is critical to initiate the extrinsic coagulation cascade in response to vascular endothelial disruption and enhances cell proliferation and migration [2].

The alternatively splice isoform of TF was identified in 2003. As this isoform is a splice variant, it was named alternatively spliced tissue factor (asTF). Compared to flTF, asTF is translated by a truncated mRNA transcript that lacks exon 5. Exon 5 of TF contains an exonic splicing enhancer (ESE) sequence motif, which can bind to the serine/arginine-rich proteins alternative splicing factor/pre-mRNA-splicing factor SF2 (ASF/SF2) and serine-rich protein55 (SRp55), leading to the generation of flTF mRNA and translation of the flTF isoform protein [3]. The fusion of exon 4 and 6 creates a frameshift mutation and leads to a unique C-terminus, which enables asTF to be soluble and be secreted into extracellular fluids [4]. The coagulation activity of asTF has been debated since it was identified. Because asTF retains the conserved residues Lys165 and Lys166 which are important for substrate recognition during TF/factor VII activated (FVIIa) complex formation, some researchers believe that asTF maintains the factor X activated (FXa) generation ability and promote coagulation. Indeed, its presence in thrombi was demonstrated [4]. TNF-α and IL-6 enhanced TF-induced coagulation in human umbilical venous endothelial cells (HUVECs) [5]. However, the location on a phospholipid membrane, a prerequisite for efficient macromolecular substrate binding, was abolished by the soluble C-terminus of asTF, which may result in the disability of its pro-coagulant effect. Meanwhile, the experimental methods used in those studies did not exclude the possibility that the coagulant activity might be due to flTF indirectly, since it is extremely difficult to distinguish the precise role of two TF isoforms in coagulation in pro-coagulant assay [6]. Moreover, in FX activation assay, the cell lysate of asTF_FLAG-transfected HEK293 cells could not lead to FX activation, while flTF_FLAG-transfected HEK293 cells showed significant conversion of FX to FXa [7]. To date, no tissue and/or naturally occurring biological settings have been described that asTF is present without the full length isoform flTF [8] new approaches with higher sensitivity and specificity are needed for this scientific issue.

In 1865, Armand Trousseau first described thrombophlebitis (also known as Trousseau’s syndrome) as a complication of pancreatic cancer. Since then, the idea that TF is involved in cancer development, including cell proliferation, survival, angiogenesis, epithelial-to-mesenchymal transition (EMT), and metastasis, has been gradually accepted [4],[9]-[15]. In some malignant cancer systems, elevated TF expression can be detected in the serum as well as in tumor tissues [16]-[18]. In addition, tumor-derived TF-positive microparticles (TF^+^-MPs) are abundant in the plasma of patients with advanced diseases [19]-[21], which also highly correlates with venous thromboembolism (VTE) [22],[23]. These findings indicate that targeting TF have potential significance for tumor diagnosis and therapy.

In this review, we shall overview the current understanding of the regulation and functions of TF in different stages of cancer progression. TF-related complications in tumor patients and TF-targeted therapy in clinical trials will also be discussed.

### Sources of TF and their regulation in cancer

Ectopic expression of TF has been detected in several type of cancers, including cervical cancers [18], epithelial ovarian cancer (EOC) [24], breast cancer [25], brain tumors [26], pancreatic cancer [27], gastric cancer [28], prostate cancer [29], colorectal cancer (CRC) [30], lung cancer [31], melanoma [32], and several cancer cell lines, including human promyelocytic leukemia tumor cell lines HL-60, glioma cell line U343, gastric cell line KATOIII, SNU-5 and MKN-74, colon cancer line HCT116, epidermoid carcinoma cell line A431, melanoma cell line WM1341B and WM938A [4],[33]. In addition, endothelial cells of tumor blood vessels, fibroblast and inflammatory cells also express TF [34],[35]. Cervical tumors, pancreatic cancer and breast cancer specimens expressed asTF in both tumor cells and the stroma [12],[36],[37]. Two distinct forms of flTF, membrane-bound flTF [38] and TF^+^-MPs [39], are important for malignancy progression. Both tumor cells and monocytes are the main sources of TF^+^-MPs. Platelets and neutrophils also contribute to the production of TF^+^-MPs [19]. For the detail cell source of TF, see online GEO database (GSE3239).

**Figure 1:**
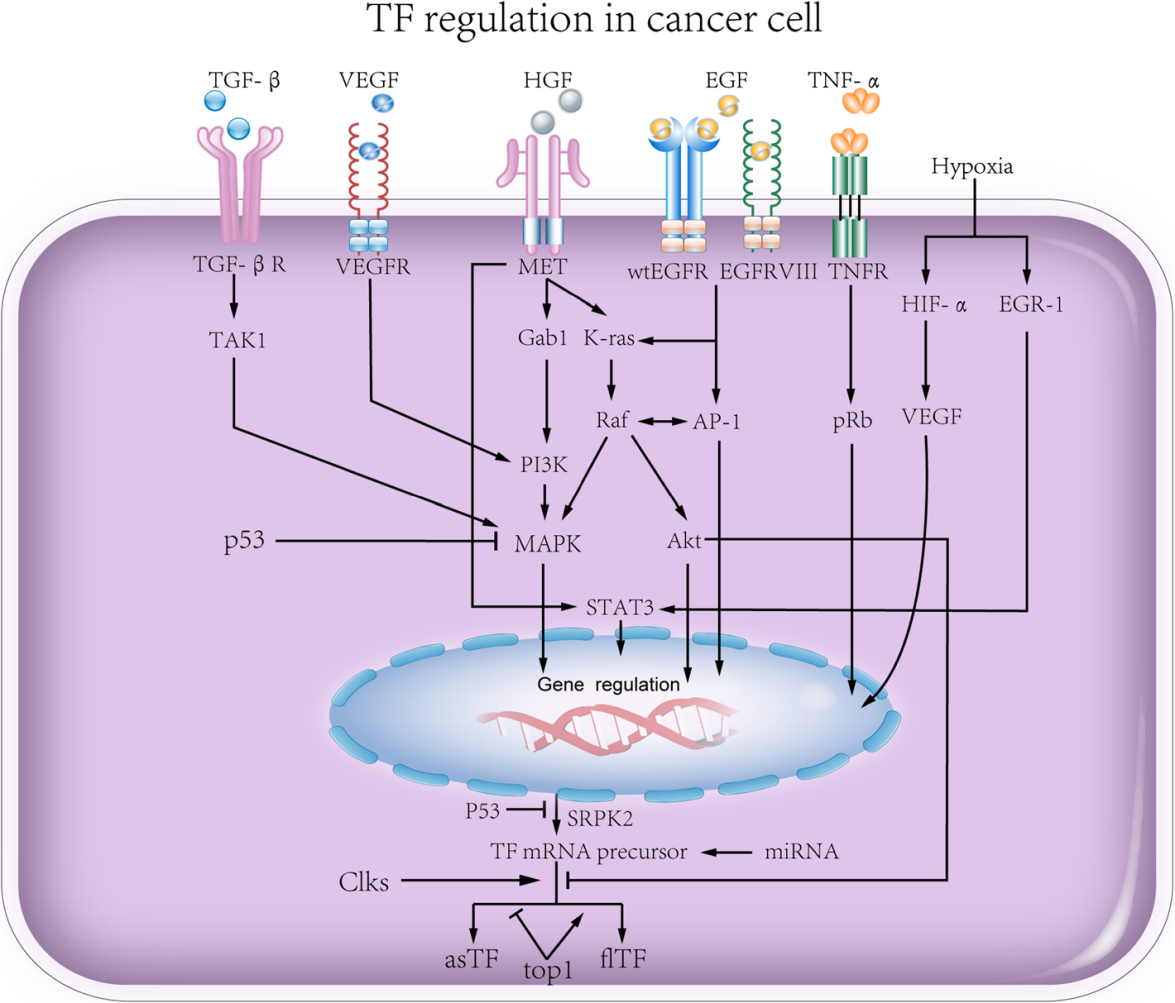
**Signaling pathways involved in TF expression.** TGF-β, VEGF, HGF, EGF, TNFα, and hypoxia challenge as well as p53 each regulates TF transcription and translation.

Given the aberrant TF expression in tumor cells, oncogenic signaling pathways participate in TF regulation (Figure 1). Evidence from *in vivo* experiments and clinical data revealed that the proto-oncogene K-ras and mutation of the tumor suppression gene p53, are primarily responsible for the upregulation of flTF. The loss function of p53 or activation of K-ras results in the activation of mitogen-activated protein kinase (MAPK)/phosphoinositide-3 kinase (PI3K) signaling pathway and subsequent induction of flTF expression [40],[41]. In squamous cell carcinoma and brain tumors, epidermal growth factor receptor (EGFR) and its mutant form EGFRvIII also regulate the expression of flTF, FVII, protease-activated receptor 1 (PAR1) and PAR2 [42]. Additionally, EGFR can activate TF transcription *via* activator protein-1 (AP-1), thus further increases TF expression [43]. AsTF expression is modulated by SF2/ASF and SRp75 through the PI3K/Akt pathway [44]. c-MET-Src family kinases are required for hepatocyte growth factor (HGF)/scatter factor induced TF expression in medulloblastoma cells. Mutation of c-MET leads to the anti-apoptotic response and resistance to chemotherapy [45]. Retinoblastoma protein (Rb), which can be induced by TNF-α [46], is an important oncogenic element leading to the aberrant expression patterns and proliferation of cancer cells [47]. flTF can be significantly upregulated in retinoblastoma cells expressing mutant pRb, a member of Rb gene family [48]. In addition, TNF-α, interferon-gamma (IFN-γ), early growth response gene-1 (EGR-1), hypoxia-inducible factor 1 alpha (HIF-1α), and transforming growth factor-beta (TGF-β) upregulate flTF in cancer cells and endothelial cells [6],[49]-[51]. TNF-α induces both TF isoform expression in HUVEC. Interestingly, this TNF-α-induced TF expression can be reduced by CDC-2 like kinases (Clks) inhibitor [52], whereas DNA topoisomerase I inhibition upregulates asTF and reduces flTF expression [6]. Moreover, microRNAs also involved in TF posttranscriptional regulation [53],[54]. Inhibition of miR-19a or miR-126 induces the expression of both TF isoforms, asTF and flTF, in endothelial cells under normal as well as under inflammatory conditions, thereby reduces the flTF-mediated pro-coagulant activity of these cells [53]-[55]. Moreover, miR-19b and miR-20a, for instance, play a role in flTF regulation in colon cancer and SLE [56],[57]. In medulloblastoma, flTF expression is accompanied by miR-520 g silencing, and overexpression of miR-520 g suppresses flTF levels [58]. More details about the regulation of the TF isoform expression were reviewed by Leppert *et al*. [59].

Collectively, TF is universally expressed in tumor cells, immune cells and stromal cells. Its overexpression in tumors suggests a potential marker and therapeutic target for cancer. Understanding the roles of TF in cancer could potentially improve our knowledge of carcinogenesis.

### Functions of TF in tumor progression

**Figure 2:**
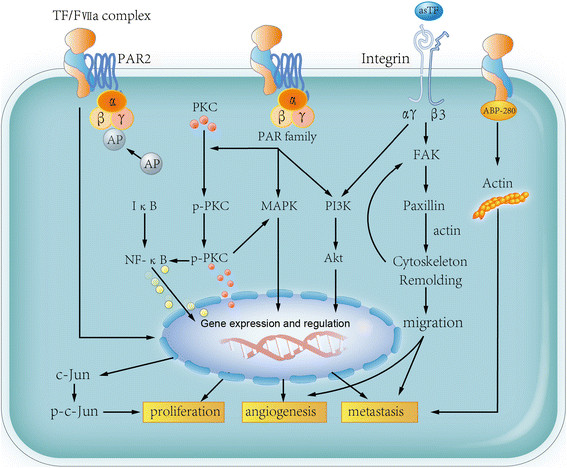
**Function of TF in cancer progression.** flTF forms TF/FVIIa complex and subsequently induce PAR signaling. PKC is phosphorylated by activated PAR complex, which leads to p-PKC translocation. PAR can also induce MAPK and PI3K activation, both of which trigger pro-tumor effects, such as proliferation, angiogenesis and metastasis. Binding to activator protein (AP) can also induce c-Jun upregulation and in turn promote tumor progression. Moreover, flTF binding to ABP-280 leads to actin modulation, resulting in tumor cells metastasis. asTF binds to integrin receptor and enhance the ability of migration, in turn leading to tumor cell angiogenesis and migration.

**Figure 3:**
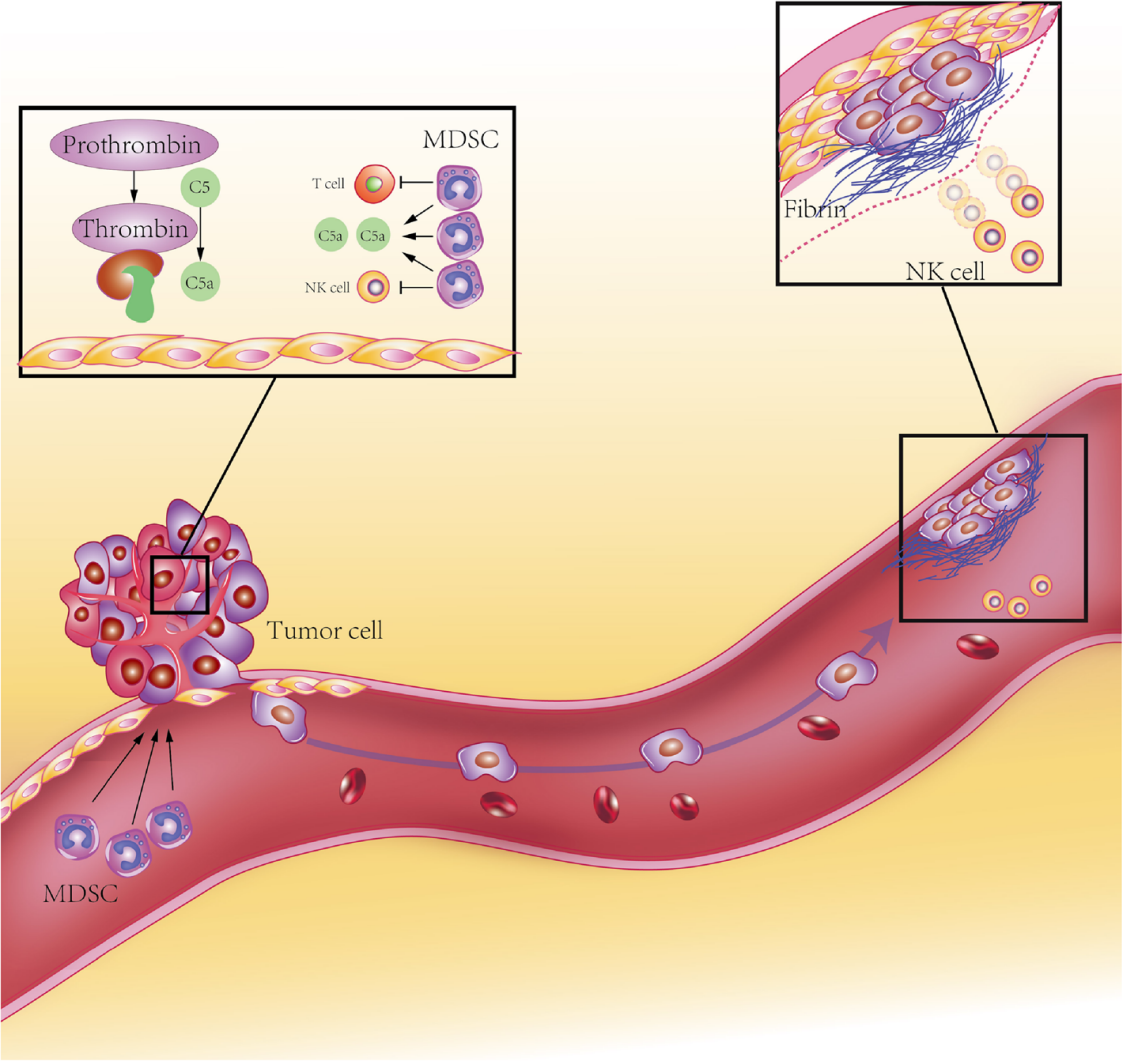
**Cancer cells escape from T/NK cell immunity*via*flTF.** flTF expressed on tumor cell surface tiggers local coagulation cascase, leading to thrombin gerneration. Thrombin induce C5 cleavage and C5a gerneration. C5a recruits MDSCs into tumor microenvironment and suppress T cell and NK cell anergy. Micrometastasis of tumor cells generates fibrin shield *via* flTF-induced coagulation, thereby preventing NK cell-induced cytolysis.

Downstream events of TF activation include thrombin generation, fibrin deposition, platelet activation, tumor-associated macrophage (TAM) recruitment, and metastasis *via* EMT [60]. Here, we mainly focus on TF functions in four aspects of cancer: sustaining proliferating signaling, resisting cell death, activating invasion and metastasis, avoiding immune destruction, and lethiferous clinical complications such as VTE (Figures 2 and 3).

### TF regulates tumor cell proliferation and apoptosis

flTF and asTF promote tumor cell proliferation through different mechanisms (Figure 2) [12],[61]. flTF/FVIIa complex can activate PAR2, leading to AP-1 phosphorylation, cell proliferation and migration in the colon cancer SW620 cell line [62]. Furthermore, activation of PAR2 by flTF induces protein kinase Cα (PKCα) phosphorylation and translocation from the cytoplasm to the perinuclear region, promotes ERK1/2 and NF-κB phosphorylation [61]. Breast cancer cell apoptosis can be suppressed by flTF *via* PI3K/Akt signaling pathway and reducing IL-8 and death-associated protein kinase 1 (DAPK1) [63]. The variant isoform asTF also promotes tumor growth in pancreatic and lung cancer setting [31],[64],[65]. Different from flTF, asTF enhances tumor cell proliferation through integrin signaling [12] which was also reviewed in detail by Leppert *et al.* in 2014 [59].

### TF promotes tumor angiogenesis and metastasis

Blood vessels in tumor tissues are essential for tumor progression, and neovasculature is a prerequisite for blood-borne metastasis. In primary breast cancer cells, flTF/FVIIa/PAR2 induces the production of pro-angiogenic factors and immune regulators [66]. Meanwhile, evidence from Hobbs *et al.* demonstrated that nude mice carrying asTF-overexpressing pancreatic ductal adenocarcinoma cells developed significantly larger tumors and increased angiogenesis than flTF-overexpressing cells [65]. asTF enhances pro-angiogenesis and pro-migration ability of cardiac cells *via* inducing angiogenesis- and migration-promoting factors such as fibroblast growth factor 2 (FGF2), cysteine-rich 61 (Cyr61) and vascular endothelial growth factor (VEGF). Meanwhile, monocytic THP-1 cells exhibit enhanced migration after treated with the supernatant of asTF-overexpressing mouse cardiomyocytic HL-1 cell [11]. Hypoxia exposure induces asTF expression in A549 cells through alternative splicing factors Clk1 and Clk4. The elevated asTF promotes the tube formation of A549 cells by increasing Cyr61, CC chemokine ligand (CCL2) and VEGF [31]. Different from flTF-PAR interaction, asTF possesses its potent pro-angiogenic properties through interacting with integrin β1 and β3 in endothelial cell, eliciting focal adhesion kinase (FAK), p42/p44, p38 MAPK and Akt phosphorylation [36]. 6B4, an antibody which disrupts the TF-integrin interaction, can efficiently inhibit the pro-angiogenic function of asTF [67].

In addition to the pro-angiogenic effects in cancer, TF also regulate cytoskeleton remodeling, which enhances tumor cell migration and subsequently promotes metastasis. flTF stimulates tumor cell migration through cytoplasmic domain by activating p38 in a Rac 1 dependent manner [68]. Specific interaction between the flTF cytoplasmic domain with actin-binding protein 280 (ABP-280) also contributes to tumor cell metastasis and vascular remodeling [69]. However, flTF exhibits its pro-metastatic characteristics mainly by initiating the pro-coagulant cascade, including thrombin formation, fibrin generation and platelet activation [70],[71]. The fibrin (ogen)-platelet clot formation is essential for generating a shield around tumor cells to facilitate the spread of tumor cells and the escape of newly formed micrometastasis from natural killer (NK) cell-mediated cytolysis [72],[73]. TFPI, an inhibitor of TF, can significantly reduce the metastasis of B16F10 murine melanoma cells [74]. The TF-induced coagulation can promote TAMs recruitment and the establishment of the pro-metastatic niche [75].

Cancer stem cells (CSC), which express CD133 [76], CD44, ATP-binding cassette sub-family G member 2 (ABCG2) and Aldehyde dehydrogenases (ALDH) [77], are a subpopulation of tumor cells that display self-renewal and the ability to give rise to heterogeneous lineages of cancer cells. These heterogeneous cells are responsible for tumor initiation, angiogenesis, and metastasis. Results from our lab revealed that CD133^+^ ovarian cancer stem cells remarkably over express flTF compared with CD133^−^ cancer cells [78]. Moreover, evidence from Chloe C. Milsom and her colleagues demonstrated that the TF-blocking antibody (CNTO 859) delays A431 cell initiation and metastasis through blocking EMT [79]. The functions of TF in angiogenesis and metastasis as well as the location of CSCs in the perivascular niche suggest that the interfering with CSCs by targeting TF would be of interest and worth for further research.

Hence, the expression of TF can effectively enhance angiogenesis and coagulation-associated metastasis *via* either the interaction of the cytoplasmic domain with the PAR family, or through the integrin signaling pathway (Figures 2 and 3).

### TF modulate immune responses within the tumor microenvironment

Cytotoxic T lymphocytes and NK cells are the major effector cells mediating anti-tumor immunity. However, anti-tumor immunity is abrogated primarily due to the dysfunction of cytotoxic T lymphocytes and NK cells and the accumulation of myeloid-derived suppressor cells (MDSCs) in the tumor microenvironment [80]. As mentioned above, flTF is responsible for local thrombin generation and fibrin deposition. Once thrombin is generated, it can directly cleaves complement component C5 to produce C5a and C5b [81]. C5a, also known as anaphylatoxin, has a pro-tumor effect *via* recruiting MDSCs to the tumor microenvironment, resulting in an immunosuppressive milieu [82] (Figure 3). Meanwhile, TF-mediated thrombosis within the tumor microenvironment may cause local ischemia and hypoxia, leading to the local inflammatory response and tumor tissue necrosis. The TF-induced hypoxia could in turn upregulate flTF, Clk1 and Clk4, resulting in asTF production [31]. This potential positive feedback loop may contribute to tumor cell proliferation and angiogenesis, as well as increase MDSC infiltration within the tumor microenvironment. TF-triggered tumor cell-clot formation induces vascular cell adhesion molecule-1 (VCAM-1) expression and the recruitment of myeloid cells, and promotes tumor invasion and metastasis [83]. Taken together, TF assists tumor cells to metastasis and escape from the host immune system *via* modulating the tumor microenvironment.

### TF expression correlates with increased VTE

Since VTE, particularly deep venous thrombosis of the lower extremities and pulmonary embolism, comprises the second leading cause of death in cancer patients [84], efficient anticoagulation therapies are of profound clinical importance. Clinical studies indicate that administration of low molecular weight heparins (LMWH) in cancer patients significantly improves survival [85]-[87].

The phosphatidylserine (PS) acts synergistically with flTF to amplify its role as a coagulation initiator [21],[88]. Both flTF and PS in systemic circulation assemble on the surface of MPs from tumors, resulting in the formation of the coagulation complex. Therefore, circulating tumor cell-derived TF^+^-MPs may trigger venous thrombosis formation in the absence of vessel injury. TF^+^-MPs in the systemic circulation of patients with advanced colorectal cancer increased the risk of VTE by two fold when compared with healthy controls [89]. Another study showed that cancer patients suffering from VTE had a higher level of TF^+^-MPs compared with those without VTE [90]. In addition to the plasma antigen level, an increase of TF^+^-MPs activity in cancer patients with VTE was reported by several groups. Tessellar *et al.* observed a higher level of TF^+^-MPs activity in acute VTE patients than in patients without VTE [91],[92]. Owens and Mackman found elevated MP-TF activity in 9 of 11 patients [19]. Similarly, Zwicher *et al.* reported a 7-fold increased risk of thrombosis in VTE-free patients with elevated TF^+^-MP levels than in VTE-free plus TF^+^-MPs negative patients [93]. The association between mortality and the level of TF^+^-MPs was also demonstrated. Tesselaar and Bharthuar individually reported that in breast cancer and pancreaticobiliary cancer, patients with VTE, who presented higher level of MP-TF activity, had a lower survival rate than patients with lower levels of MP-TF activity [23],[91]. These studies indicate that TF^+^-MP amount and MP-TF activity may have prognostic values in cancer patients.

## Conclusion and prospective

In conclusion, the traditional extrinsic coagulation pathway initiator flTF and its isoform actively participate in malignant disease progression. The signaling pathways associated with TF are critical for tumor initiation, growth, angiogenesis and metastasis and clinical complications such as VTE. Targeting flTF and anticoagulation therapies have already been used for several types of cancer [26]. Understanding the precise regulatory mechanisms of flTF as well as its soluble isoform asTF in tumor progression could be of potential interest for improving the theory of tumor immunoediting and developing individual therapeutic strategies for cancer.

## Abbreviations

ABP-280: Actin-binding protein 280

ABCG2: ATP-binding cassette sub-family G member 2

ALDH: Aldehyde dehydrogenase

AP-1: Activator protein-1

APC: Activated protein C

APL: Acute promyelocytic leukemia

ASF/SF2: Alternative splicing factor/pre-mRNA-splicing factor SF2

asTF: Alternatively spliced tissue factor

ATRA: All-trans retinoic acid

CCL2: CC chemokine ligand

Clk: CDC-2 like kinases

CRC: Colorectal cancer

CSCs: Cancer stem cells

Cyr61: Cysteine-rich 61

EGFR: Epidermal growth factor receptor

EGR-1: Early growth response gene-1

EMT: Epithelial-to-mesenchymal transition

EOC: Epithelial ovarian cancer

ESE: Exonic splicing enhancer

FAK: Focal adhesion kinase

FGF: Fibroblast growth factor

flTF: Full-length tissue factor

HGF: Hepatocyte growth factor

JNK: c-Jun amino-terminal kinase

LMWH: Low molecular weight heparins

MAC: Membrane attack complex

MAPK: Mitogen-activated protein kinase

MDSCs: Myeloid-derived suppressor cells

MMP-1: Matrix metalloproteinase-1

MPM: Malignant pleural mesothelioma

NSCLC: Non-small cell lung cancer

OCSC: Ovarian cancer stem cells

PAK-1: p21-activated kinase 1

PAR: Protease-activated receptor

PDGF: Platelet-derived growth factor

PI3K: Phosphoinositide-3 kinase

PKCα: Protein kinase Cα

PS: Phosphatidylserine

RAS: Renin-angiotensin system

TAMs: Tumor associated macrophages

TF: Tissue factor

TF^+^-MPs: Tissue factor-positive microparticles

TFPI: Tissue factor pathway inhibitor

TICs: Tumor-initiating cells

TM: Thrombinmodulin

VCAM-1: Vascular cell adhesion molecule-1

VEGF: Vascular endothelial growth factor

VTE: Venous thromboembolism

## Competing interests

The authors declare that they have no competing interests.

## Authors’ contributions

XH, YL and BZ drafted the manuscript and figures; YL, BG and BZ contributed to editing of the manuscript. All authors have read and approved the final manuscript.
